# Systems pharmacology based approach to investigate the in-vivo therapeutic efficacy of Albizia lebbeck (L.) in experimental model of Parkinson’s disease

**DOI:** 10.1186/s12906-019-2772-5

**Published:** 2019-12-05

**Authors:** Uzma Saleem, Zohaib Raza, Fareeha Anwar, Zunera Chaudary, Bashir Ahmad

**Affiliations:** 1 Department of Pharmacology, Faculty of Pharmaceutical Sciences, Government College University, Faisalabad, Pakistan; 2 Riphah Institute of Pharmaceutical sciences, Riphah International University, Lahore, Pakistan

**Keywords:** System pharmacology, PD, Haloperidol, Albizia lebbeck

## Abstract

**Background:**

Parkinson’s disease (PD) is a progressive neurodegenerative disorder characterized by loss of dopaminergic neurons in substantia nigra pars compacta and clinically manifested mainly with motor dysfunctions. Plants are rich source of medicinally important bioactive compounds and inhabitants of underdeveloped countries used plants for treatment of various ailments. *Albizia lebbeck* has been reported to possess antioxidant and neuroprotective properties that suggest the evaluation of its traditional therapeutic potential in neurodegenerative diseases.

The aim of present study was to validate the traditional use of *Albizia lebbeck* (L.) and delineate its mechanism of action in PD. The systems pharmacology approach was employed to explain the *Albizia lebbeck* (L.) mechanism of action in PD.

**Methods:**

The haloperidol-induced catalepsy was adopted as experimental model of PD for in-vivo studies in wistar albino rats. The systems pharmacology approach was employed to explain the *Albizia lebbeck* (L.) mechanism of action in PD.

**Results:**

In-vivo studies revealed that *Albizia lebbeck* improved the motor functions and endurance as demonstrated in behavioral studies which were further supported by the rescue of endogenous antioxidant defense and reversal of ultrastructural damages in histological studies. System pharmacology approach identified 25 drug like compounds interacting with 132 targets in a bipartite graph that revealed the synergistic mechanism of action at system level. Kaemferol, phytosterol and okanin were found to be the important compounds nodes with prominent target nodes of TDP1 and MAPT.

**Conclusion:**

The therapeutic efficiency of *Albizia lebbeck* in PD was effectively delineated in our experimental and systems pharmacology approach. Moreover, this approach further facilitates the drug discovery from *Albizia lebbeck* for PD.

## Background

Parkinson’s disease (PD) is a chronic age-related neurodegenerative disorder characterized mainly with motor dysfunctions including resting tremors, muscular rigidity, bradykinesia and postural reflex impairments. It has been prevalent in 10 million people around the globe with incidence rate of 219/100000 people in Pakistan [1]. There has been evidence that suggests the oxidative stress, accumulation of misfolded protein and the loss the dopaminergic neurons in substantia nigra pars compacta as the main hallmarks of PD pathogenesis [2]. The neurodegeneration has been accounted for the loss of 80% dopaminergic neurotransmission in striatum that leads to significant neuromuscular dysfunction along with some cognitive deficits at advanced stages [3]. Levodopa is the primary gold standard approach to symptomatically manage the PD but its chronic use has also been associated with development of dyskinesia [4]. Moreover, we have no therapeutic options that provides the neuroprotection or relieve the progression of PD. Therefore, it is the need of time to develop the therapeutic modalities that changes the course of PD progression along with treating it symptomatically.

Plants have been increasingly reported to possess the diverse range of therapeutic compounds in the development of therapeutic options for PD [5]. However, it is costly and tedious to characterize the therapeutic roles of a plant species to the diverse or complex nature of bioactive phytochemicals present in it. Recently, the approach of systems pharmacology has been emerged as a sophisticated tool to dissect the complex pharmacological behavior of phytochemicals within the plant species. This approach integrates the high-dimensional biological data for phytochemicals to construct the computational model to explain their synergism in the development of pharmacological space at the biological systems level. The system pharmacology model extrapolates the phytochemicals from molecular or cellular to organism level in the form of biological network that better explain the mechanism of action and potential drug development from plant species [6]. *Albizia lebbeck* (L.) *Benth*, commonly known as Siris, is a large deciduous tree native to tropical southern Asia, widely cultivated in other tropical and subtropical regions. It is a tree with bi-pinnate leaves and white fragrant flowers with a fruit that is a pod containing six to twelve seeds [7]. Traditionally, pods and seeds have been widely used as antiprotozoal, anti-asthmatic, anticancer, antidiabetic, antidiarrheal, aphrodisiac and as a brain tonic [8]. The alcoholic extract of *Albizia lebbeck* (L.) has been reported to possess antihistaminic property, by neutralizing histamine directly or due to corticotrophic action [9]. Alcoholic extract of the stem bark of *Albizia lebbeck* (L.) was further corroborated to possess the strong analgesic and moderate anti-inflammatory activities possibly due to the presence of steroids and steroidal glycosides [10]. A study suggested that 70% ethanolic extract of bark of *Albizia lebbeck* (L.) also possess antioxidant and hepatoprotective effects owing to its principle phenolic components [11]. Moreover, the aqueous extract of *Albizia lebbeck* (L.) was found to reduce oxidative stress in alloxan-induced diabetic rats [12]. *Albizia lebbeck* (L.) bark has been found to be involved in disturbed testicular somatic cell function owing to its antifertility potential [13]. *Albizia lebbeck* is a diverse source of bioactive secondary metabolites including the higher content of saponins, vitamins and polyphenolics or flavonoids owing to its potential antioxidant activity or may be the therapeutic properties [14–16]. The polyphenolics have also been widely conferred with therapeutic potential to fix the redox balance and induction of endogenous antioxidant defense [17, 18]. There are mounting evidences that addresses the role of oxidative stress to regulate the dopamine metabolism, neuro-inflammation and neuronal loss in Parkinson’s [19]. Moreover, *Albizia lebbeck* has also been found to modulate the locomotion and motor coordination of experimental convulsive rats [20]. Despite its potent antioxidant and neuro-modulatory capacity, the neuropharmacological studies of *Albizia lebbeck* (L.) has been limited to anticonvulsant, antidepressant and anxiolytic activities with no direct evidence related to major neurodegenerative disorder i.e. Parkinson’s [20–22]. These insights provide reasonable justifications to further evaluate the traditional use of *Albizia lebbeck* in neurodegenerative disorders. Therefore, this study is aimed to employ the In-vivo and system’s pharmacology approach to validate the traditional use and investigate the mechanism of action of the *Albizia lebbeck* (L.) in Parkinson’s disease.

## Methods

### Experimental validation

#### Chemicals

Methanol, Trichloroacetic acid (TCA), 5,5′-dithio-bis(2-nitrobenzoic acid) (DTNB), Hydrogen Peroxide (H_2_O_2_), potassium dihydrogen phosphate, pyragallol, potassium hydroxide, di-potassium hydrogen phosphate were purchased from Sigma-Aldrich (USA). Ketamine was obtained from Caraway pharmaceutical (Pakistan). Sinemet was obtained from OBS (Pakistan). All the chemicals used were of analytical grade.

#### Collection and authentication of plant material

The *Albizia lebbeck* (L.) seeds were collected from botanical garden of University of Agriculture Faisalabad (UAF), Faisalabad and authenticated by taxonomist Dr. Mansoor (UAF) with authentication voucher specimen (620–1-18) deposited in UAF Herbarium.

#### Preparation of plant material and aqueous methanolic *Albizia lebbeck* (L.) extract (ALE)

Seeds were washed with tap water, air dried, grinded by mechanical milling and sifted into a fine powder that was subjected to cold extraction. Powder material (1 kg) was cold macerated in 1:2 ratio with 80% aqueous methanolic solvent (2 L) with 12 h periodic stirring for 14 days. At the end of maceration, macerate was subjected to primary filtration through filtration cloth to obtain the supernatant filtered off the macerated powder material. The supernatant was filtered through Whatman No. 1 filter paper in secondary filtration to remove the suspended solid particles from filtrate. Subsequently, the solvent in pure filtrate was evaporated by rotary evaporator under reduced pressure at 40–45 °C that provided 9.9% extract yield percentage of pure extract.

### In-vivo anti-Parkinson’s activity

#### Ethical approval for animal studies

The experimental study (No. 19589) was performed after getting ethical approval from Institutional Review Board with reference no. GCUF/ERC/1989 ruled under the regulation of Institute of Laboratory Animal Resources, Commission on Life Sciences University, National Research Council (1996).

#### Animals and husbandry

The experimental studies were performed on healthy wistar albino rats of same age, sex and strain. The rats were purchased from University of Agriculture Faisalabad (UAF), Faisalabad and maintained on laboratory diet with water ad libitum in animal house of Government College University Faisalabad (GCUF), Faisalabad. The rats were acclimatized for one week prior to experimental studies and kept in stainless steel cages in a temperature control (24 ± 1 °C) and chemical free environment with natural light and dark cycle. Personalized human care was provided to all rats and experiments were conducted in a noiseless facility under adequate light system.

#### Experimental design

The following experimental model was followed to evaluate the pharmacological potential of *Albizzia lebbeck* (L.) in Parkinson’s disease:

- Group I: Normal control (NC);
- Group II: Disease control (HPD);
- Group III: Standard control (STD);
- Group IV: 100 mg/kg treatment control (ALE 100);
- Group V: 200 mg/kg treatment control (ALE 200);
- Group VI: 300 mg/kg treatment control (ALE 300);

Total 36 rats were employed. There were six rats (*n = 6*) weighing 100–150 g in each group.

The method of chitra et al. was used to induce the Parkinson’s in experimental rats [23]. Normal control group (Group I) received the distilled water as vehicle. The intraperitoneal injection of haloperidol (1 mg/kg) was administered to group II-VI for 21 days to induce the catalepsy in rats. However, the Group III was treated with standard (Sinemet - levodopa 100 mg + carbidopa 25 mg /kg per oral) and groups IV-VI were administered with 100, 200, 300 mg/kg per oral, respectively, doses of ALE 30 min before the haloperidol administration. At the end of treatment for 21 days, the behavioral testing of rats was conducted to assess the catalepsy and motor functions.

### Behavioral tests

#### Assessment of catalepsy

Mograbi et al., (2017) method was adopted for induction of catalepsy [24]*.* Functional assessment of catalepsy was performed by the bar method. The wooden bar (diameter 1 cm) was utilized for this purpose that was elevated 3 cm from floor. Both forelimbs of rats were placed on the bar and time (seconds) was recorded for the rats to correct their imposed position. End point of catalepsy was considered when rat’s forelimbs touched down on the floor or if they climbed on the bar. This behavioral assessment was conducted at the 30, 60 and 90 min after the treatment with second drugs (i.e. standard or ALE).

#### Hang test

Caudal et al.*,* (2018) method was adopted for wire hanging test [25]. This test is used to evaluate the skeleton muscle strength, tone and endurance as a function of time. The apparatus is made up of 1.5 mm in diameter stainless steel wire that is 10 cm and 45 cm in length and above the flat surface of apparatus respectively The rats are allowed to hang by the wire midway between the supports through their forearms and the hanging time is recorded which is proportional to the rats global muscular strength.

#### Narrow beam walk test

Chonpathomikunlert et al.*,* (2018) method was adopted for narrow beam walk test with some modifications [26]. The rats trained and trialed to walk through the wooden narrow beam (L 100 cm × W 4 cm) for 120 s to assess the motor coordination and balance of the rats. The narrow beam was attached with two opposite platforms and time latency to reach from one platform to another was recorded as a measure to motor coordination and balance in Parkinson’s.

#### Open field test

This study was performed to assess the locomotor and exploratory behavior of animals. The apparatus for this study was wooden square box (W 100 cm × D 100 cm × H 45 cm) covered with resin and floor divided into 25 squares. Rats were allowed to move freely into the box for two minutes and following parameters were observed and recorded [27]:

- Number of squares (both central & peripheral) explored (i.e. Horizontal exploration)
- Number of rearing (i.e. Vertical exploration)

After behavioral experiments, the animals were anesthetized with intramuscular injection of ketamine hydrochloride (24 mg/kg). Following anesthetization, the animals were sacrificed by decapitation to excise the brain tissue and carcass were buried. The excised brain tissues were used for histological and biochemical assessment of oxidative stress.

#### Biochemical estimation of oxidative stress in brain

The brain tissue were homogenized with 1:10 ratio in phosphate buffer (7.4 pH) and centrifuged with 600 rpm at 4 °C for 10 min. The resultant clear supernatant was used for estimation of following biochemical parameters of oxidative stress by the method described previously [28]:

#### Determination of GSH content

The supernatant (1 ml) was precipitated with 10% of TCA (1 ml) and its aliquot was further mixed with phosphate solution (4 ml) and 0.5 ml DTNB reagent. The solution was further used for spectroscopic analysis at the 412 nm to determine the GSH content by following formula:

$$ \mathrm{GSH}=\mathrm{Y}-0.00314\div 0.034\times \mathrm{DF}\div \mathrm{BT}\times \mathrm{VU} $$

Where,

DF = Dilution factor (i.e. 1);

VU = Volume of aliquot;

Y = absorbance at 412 nm;

BT = Tissue homogenate of brain (1 ml).

#### Determination of superoxide dismutase (SOD) activity

The supernatant (0.1 ml) was added with 0.1 M potassium phosphate buffer (2.8 ml, 7.4 pH) and solution was further used for spectroscopic analysis at 325 nm. The standard curve of SOD was plotted by using different concentrations (10 μL– 100 μL) and following regression equation was used to estimate the SOD activity

$$ \mathrm{Y}=0.0095\mathrm{x}+0.1939 $$

#### Determination of catalase (CAT) activity

The supernatant (0.05 ml) was mixed with 30 mM hydrogen peroxide (1 ml) and 50 mM phosphate buffer (1.95 ml, pH 7) and resultant solution was subjected to spectroscopic analysis at 240 nm to estimate the CAT activity by following formula [29]:

$$ \mathrm{CAT}\ \mathrm{activity}=\updelta \mathrm{OD}\div \mathrm{E}\times \mathrm{volume}\ \mathrm{of}\ \mathrm{sample}\ \left(\mathrm{ml}\right)\times \mathrm{mg}.\mathrm{of}\ \mathrm{protein} $$

Where:

δOD = Changing absorbance / minute.

E = Extinction coefficient of hydrogen peroxide (i.e. 0.071 mmol cm^− 1^).

Standard curve of protein was plotted by using different concentration of BSA. Following regression equation was used to calculate the protein content.

Y = 0.00007571x + 0.0000476.

#### Histological studies

The brain tissue was fixed in 4% formaldehyde and embedded in paraffin. Then tissue was sliced into 5 μm sections and stained with Hematoxylin and Eosin (H&E) to examine under light microscopy [27].

#### Statistical analysis

The results are presented as mean ± SEM. Data were analyzed by applying One-way and Two-way ANOVA followed by tukey multiple comparison and Bonferroni posttests, respectively by using graphpad prism version 5. Statistically, the results with *p* ≤ 0.05 were considered significant.

#### Systems pharmacology of *Albizia lebbeck* (L.)

The phytochemicals of *Albizia lebbeck* (L.) were retrieved from Indian Medicinal Plants, Phytochemistry and Therapeutics (IMPPAT) and Dr. Duke’s phytochemical and ethnobotanical database to construct the compound database [30, 31]. These compounds were screened for drug likeness as a function of Lipinski rule of 5 in Swiss ADME tool [32]. The compounds, following the Lipinski criteria, were mapped by reverse pharmacophore modelling in Swiss Target Prediction tool that utilize the knowledge-based algorithm or homology based modelling to accurately predict their target proteins [33]. The target proteins with higher estimated probability were selected to construct the target database. These proteins were further classified by Gene Ontology in Panther classification system and possible functional interactions of these targets were analyzed in STRING 10 and KEGG pathways with false discovery rate < 0.5 were selected [34, 35]. These KEGG pathways were indexed with KEGG disease database to probe their disease associations. The networks of compounds – targets (C-T network) and pathway – disease (P-D network) were constructed and analyzed by Cytoscape 3.2.1 [36].

## Results

### In-vivo anti-Parkinson’s activity

#### Behavioral tests

##### Assessment of catalepsy

**Fig. 1:**
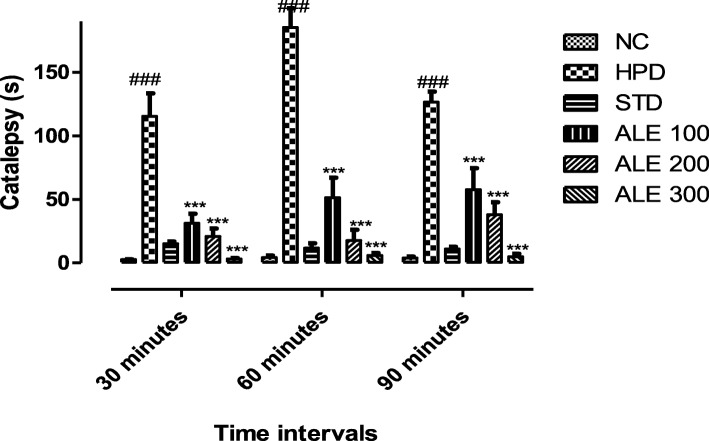
Catalepsy in time (s) at different interval; values are expressed as mean ± SEM, *n = 6* The intergroup and interinterval variation was measure by Prism two-way ANOVA followed by Bonferroni post-hoc test. ***P 0.001 vs HPD group, ^###^*P* < 0.001 vs NC (Normal control).

Animals treated with haloperidol (HPD) exhibit significantly severe catalepsy as compared to all other group (Fig. 1). The ALE 100 group revealed significant (*P* < 0.005) improvement in catalepsy. However, the ALE 200 and ALE 300 groups were more proficient in reversal of catalepsy as it results into reversal of catalepsy significantly higher (*P* < 0.001) which was comparable to STD group. Time of catalepsy was higher (185.3 ± 29.67) at the interval of 60 min that ameliorated with passage of time.

#### Hang test

**Fig. 2:**
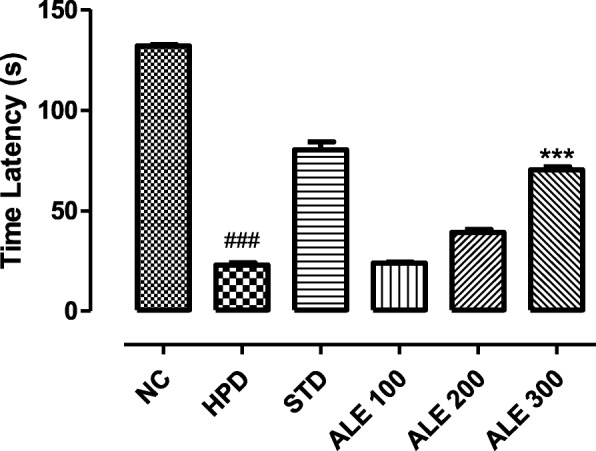
Hang time latency in experimental groups; values are expressed as mean ± SEM, *n = 6*. The intergroup variation was measure by Prism one-way ANOVA followed by tukey’s multiple comparison post-hoc test. ***P 0.001 vs HPD group, ^###^*P* < 0.001 vs NC (Normal control)

The rats treated with HPD exhibited sharp decrease in the hang time latency (Fig. 2). The treatment groups were found to increase the hang time latency in dose dependent manner. Hang time latency was higher in ALE 300 amongst the treatment group with significantly higher variation (P < 0.001) as compared to HPD group. The hang time latency of this group was also comparable to the STD group. However, the time latency of ALE 100 was moderately significant as compared to the HPD group. Taking this into consideration, dose dependent increase in hang time latency can be correlated to the global muscular strength recovery in ALE 300 rats.

#### Narrow beam walk test

**Fig. 3:**
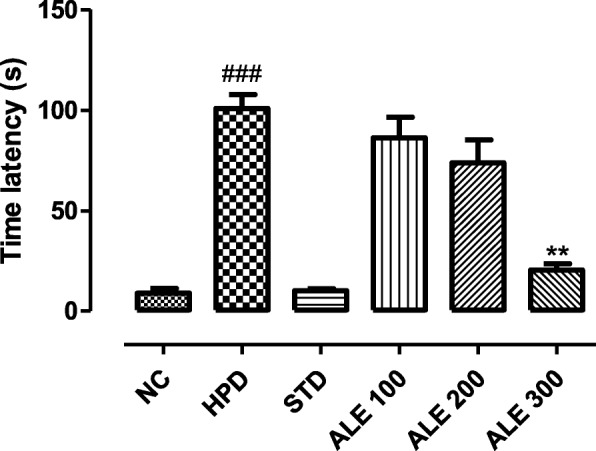
Time latency to reach the opposite platform in groups; values are expressed as mean ± SEM, *n = 6*. The intergroup variation was measure by Prism one-way ANOVA followed by tukey’s multiple comparison post-hoc test. **P 0.01 vs HPD group, ^###^*P* < 0.001 vs NC (Normal control)

The rats treated with HPD were found to exhibit motor incoordination and imbalance typically expressing the symptoms of Parkinson’s. The time latency to reach the opposite platform was higher in the group treated with HPD alone as compared to NC group (*P* < 0.001) (Fig. 3). The time latency was much lower in STD with significant variance *P* < 0.001. The difference in time latency of ALE 100 and ALE 200 was non-significant (*P* > 0.005) as compared HPD group that represented the motor intoxication in these groups. The ALE 300 group, however, revealed the highly significant variance (*P* < 0.001) as compared to HPD group and non-significant variance (P > 0.005) with standard group. Therefore, the rate of recovery from motor imbalance or intoxication was almost equivalent to the standard group.

#### Open field test

**Fig. 4:**
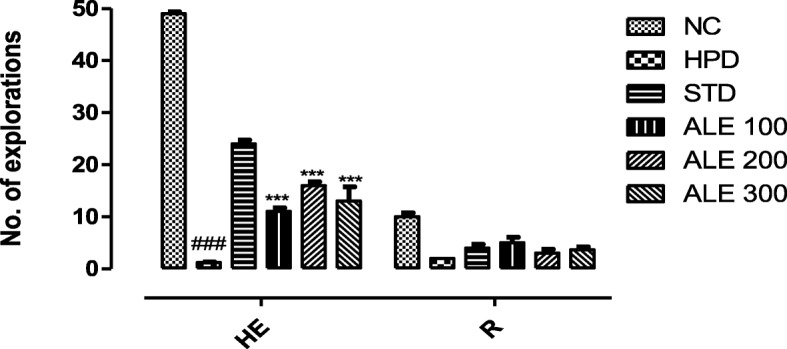
Horizontal exploration and rearing among the groups; values are expressed as mean ± SEM, *n = 6*. The intergroup variation was measure by Prism two-way ANOVA followed by Bonferroni post-hoc test. HE = Horizontal exploration, R = Rearing. ***P 0.001 vs HPD group^, ###^*P* < 0.001 vs NC (Normal control)

The HPD treated animal exhibited the marked decrease in horizontal explorations resulting into lower locomotor activities and explorations (Fig. 4). The horizontal exploration or squares crossing were recovered in the treatment group. The highest horizontal explorations in treatment groups (16 ± 0.73) were seen in the treatment group ALE 200 with highly significant variation (*P* < 0.001). However, this standard group exhibited more reversal of horizontal explorations than all the treatment groups. The rearing shared non-significant variation (*P* < 0.005) among the group.

#### Biochemical estimation of oxidative stress in brain

The antioxidant enzymatic activities in rats brain tissues was assessed to measure the status of oxidative stress following induction of Parkinson’s (Table 1). The content of GSH was severely depleted in HPD group as compared to NC group (*P* < 0.001). The recovery of GSH content was highly significant in treatment group ALE 300 which was comparable to replenishment of GSH in STD group (*P* < 0.005). However, the treatment groups ALE 100 and ALE 200 also showed significant recovery of GSH content as compared to HPD group (P < 0.001). The enzymatic activity of SOD and CAT was compromised in HPD group that resulted into oxidative stress and depletion of GSH. The recovery of enzymatic activity of CAT was moderately significant in ALE 100 and ALE 200 groups (P < 0.005). However, the recovery of CAT activity was found to be the comparable to STD group that was, in fact, highly significant rescue as compared HPD group (P < 0.001). The variance in the SOD activity was almost same in all treatment groups and STD group as compared to the HPD group (*P* > 0.005). The HPD group, however, had the lowest titer of SOD activity as compared to all groups.

**Table 1:** Estimation of endogenous anti-oxidant capacity in brain tissue of the experimental groups

Groups	Treatment	DOSE	GSH (μg/mg of brain tissue)	SOD (μg/mg of brain tissue)	CAT (μg/mg of brain tissue)
I	Normal control	N/A	80.67 ± 0.866	24.193 ± 0.6776	21.92 ± 0.487
II	Disease control (HPD)	1 mg/kg	55.624 ± 1.774	16.4716 ± 0.649	3.0733 ± 0.214
III	Standard control (STD)	100 mg/kg	79.944 ± 1.772***	22.88 ± 0.407***	13.0083 ± 0.722***
IV	Treatment control (ALE 100)	100 mg./ kg	71.29 ± 2.33 ***	21.513 ± 0.574**	11.612 ± 0.485***
V	Treatment control (ALE 200)	200 mg/ kg	77.984 ± 1.098***	22.234 ± 0.627**	16.1416 ± 0.593***
VI	Treatment control (ALE 300)	300 mg/kg	78.577 ± 1.546***	23.197 ± 0.6389***	19.55 ± 1.209***

*** P < 0.001 and ** P < 0.01 as compared to disease group. Each value is mean ± SEM

#### Histological studies

**Fig. 5:**
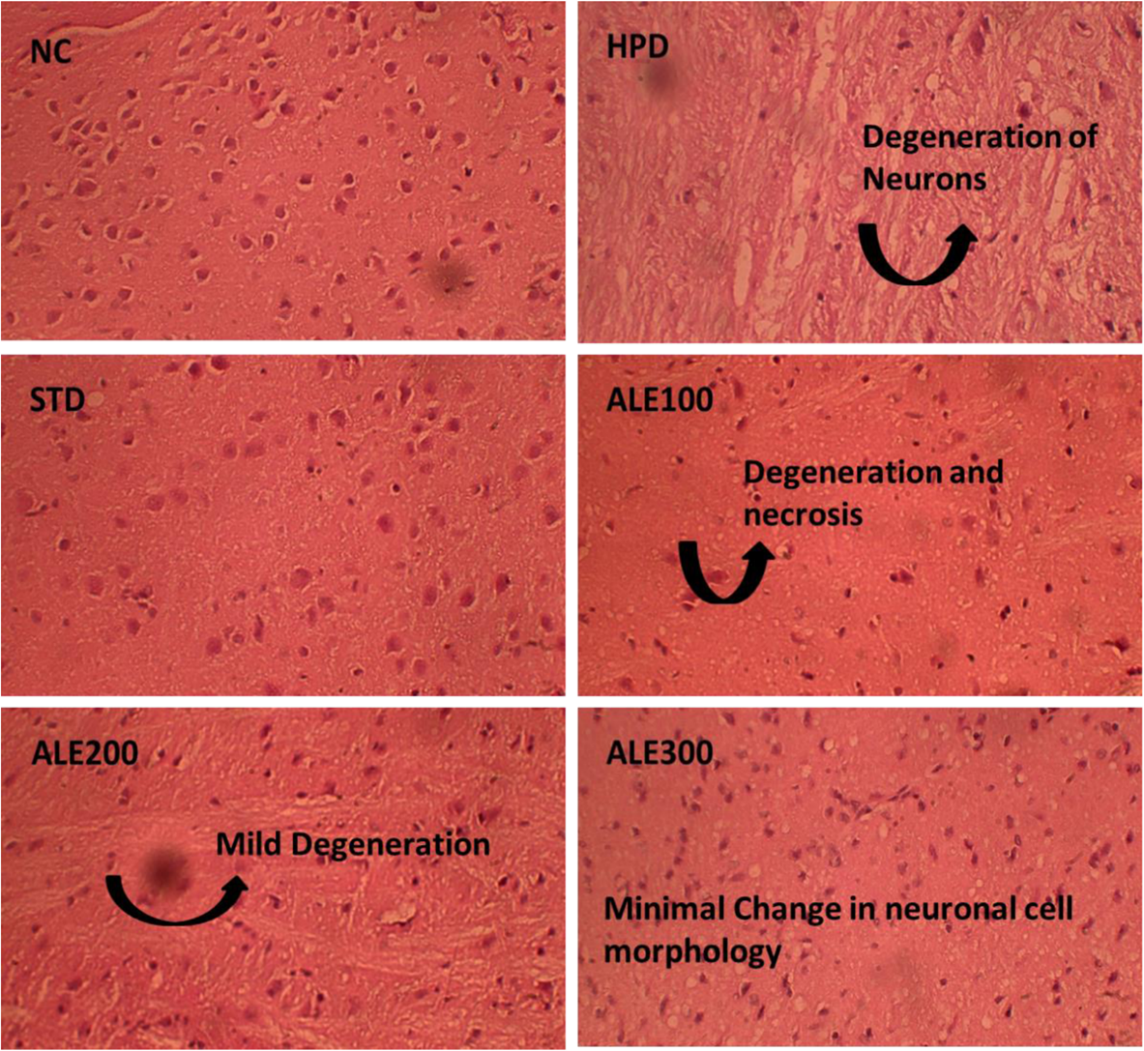
Histopathological changes in the cerebral cortex of experimental group; NC: Normal control; HPD: Disease control; STD: Standard control; ALE: *Albizia lebbeck* (L.) extract; 100, 200 & 300: dose levels in mg/kg

Histological examination of NC section revealed the intact tissue architecture with active neuronal cells (Fig. 5). The HPD section showed the severe loss of cellular outlines and chromatic content. The severe lipids peroxidation of cell membranes was accompanying with marked neurodegeneration. The STD section was significantly replenished with healthy active neurons with normal structural outlines and chromatin content. The reversal of architectural loss was also observed in ALE 100 and ALE 200 tissue sections but with the load of partially degenerated neurons with pyknotic nuclei. The ALE 300 section significantly recovered from neuronal loss and ultrastructural damages but vacuolation still persists.

#### Systems pharmacology of *Albizia lebbeck* (L.)

A total 30 representative compounds of *Albizia lebbeck* (L.) were retrieved from the database mining. The compounds were screened for logP, molecular weight, hydrogen bond donor and hydrogen bond acceptor functions as per Lipinski rule of five. All the compounds, except digitoxin, reynoutrin, vicenin 2 and rutin, passed the Lipinski criteria of drug likeness with no more than 1 violation. The list of representative compounds and their drug likeness is outlined in Table 2.

**Table 2:** The representative phytochemicals of Albizia lebbeck (L.) and their corresponding druglikeness

Compounds	Pubchem /ChEBI ID	Log P	Molecular Weight	H- donor	H-acceptor	Drug Likeness
Albigenin	Pubchem 101,280,261	6.02	426.67	1	2	Yes
Albigenic acid	Pubchem 101,596,822	5.42	472.7	3	4	Yes
Quebrachitol	ChEBI 111	−2.26	194.18	5	6	Yes
Phytosterol	Pubchem 12,303,662	7.19	414.17	1	1	Yes
Triterpenoids	Pubchem 71,597,391	3.94	472.66	4	5	Yes
Alpha Amyrin	Pubchem 225,688	7.05	426.72	1	1	Yes
Beta Amyrin	Pubchem 225,689	7.05	426.72	1	1	Yes
Epicatechin	Pubchem 182,232	0.85	290.27	5	6	Yes
Umbellic acid	Pubchem 446,611	0.89	180.16	3	4	Yes
Acacic acid	Pubchem 12,305,894	4.46	488.7	4	5	Yes
Benzoic acid	Pubchem 243	1.44	122.12	1	2	Yes
Benzyl alcohol	Pubchem 244	1.41	108.14	1	1	Yes
Celastrol	Pubchem 122,724	5.16	450.61	2	4	Yes
Digitoxin	Pubchem 441,207	2.61	764.94	5	13	No
L-arginine monocation	Pubchem 1,549,073	−3.14	175.21	4	2	Yes
Echinocystic acid	Pubchem 73,309	5.3	472.7	3	4	Yes
Eupatin	Pubchem 5,317,287	2.12	360.31	3	8	Yes
Friedlein	Pubchem 244,297	7.45	426.72	0	1	Yes
Kaempferol	Pubchem 5,280,863	1.58	286.24	4	6	Yes
Ascorbic acid	Pubchem 54,670,067	−1.28	176.12	4	6	Yes
Leucopelargonidin	Pubchem 3,286,789	0.58	290.27	5	6	Yes
Melacacidin	Pubchem 169,996	0.36	306.27	6	7	Yes
Melanoxetin	Pubchem 15,560,442	1.19	302.24	5	7	Yes
Myricitrin	Pubchem 5,281,673	−0.23	464.38	8	12	Yes
Okanin	Pubchem 5,281,294	1.69	288.25	5	6	Yes
Oleanolic acid	Pubchem 10,494	6.06	456.7	2	3	Yes
Quercetin	Pubchem 5,280,343	1.23	302.24	5	7	Yes
Reynoutrin	Pubchem 5,320,863	0	434.35	7	11	No
Vicenin 2	Pubchem 3,084,407	−2.07	594.52	11	15	No
Rutin	Pubchem 24,832,108	−1.12	610.52	10	16	No

**Fig. 6:**
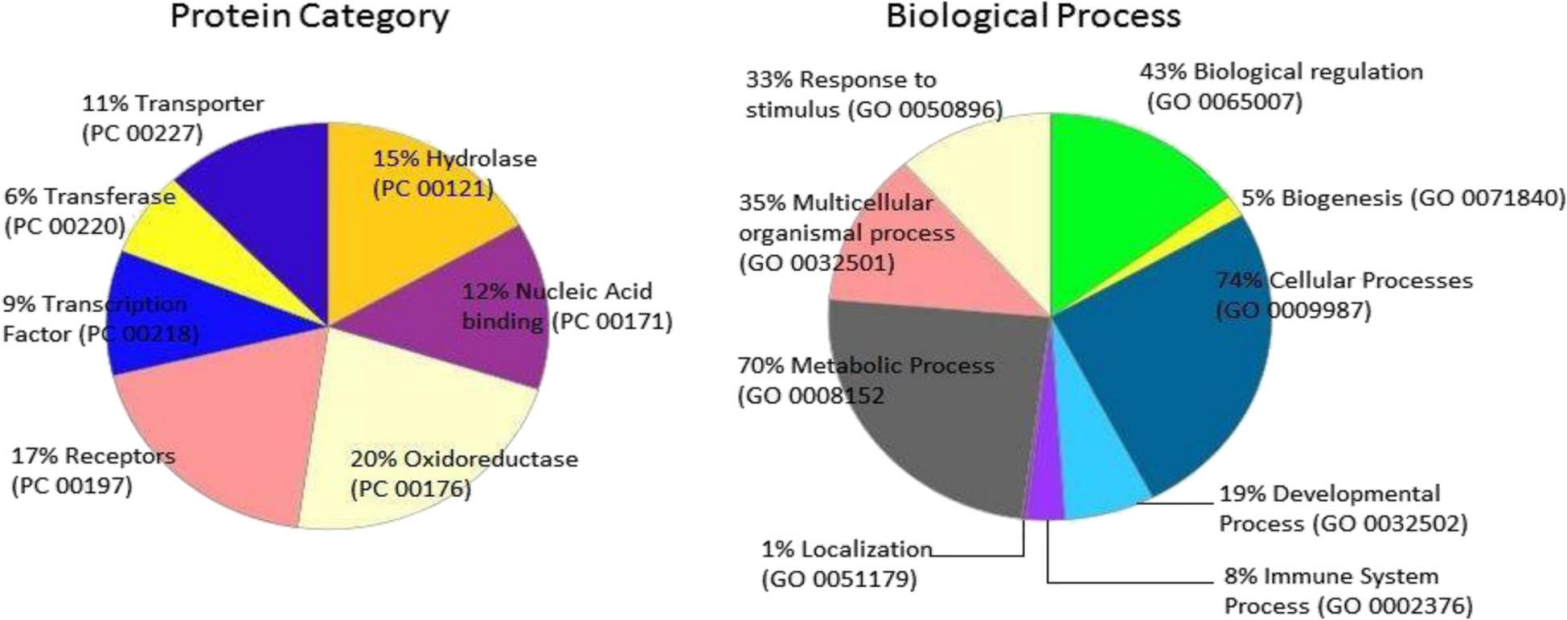
Classification of proteins by Panther classification system; Pie-Charts illustrating the percentage functional classification of proteins (left), and their involvement in biological processes (right). The protein categories and biological processes are entitled with protein classifiers (PC) and gene ontology (GO) codes, respectively

The reverse pharmacophore screening of 26 compounds, demonstrating drug likeness, yielded the 132 targets of homosapien origin except ascorbic acid. The Panther classification system classified these targets into oxidoreductase (20%), receptors (G-Protein coupled) (17%), hydrolases (15%), nucleic acid binding proteins (12%), transporter (11%), transcription factors (9%) and transferases (6%) (Fig. 6). The hydrolases were distributed predominantly by proteases (53.3%), phosphatases (33.3%), lipases (13.3%) and phosphodiesterases (6.7%). The transferases, transcription factors and transporter were predominantly distributed into the subgroups of kinases (100%), zinc finger transcription factor (77.8%) and cation transporters (63.3%), respectively. The panther classification analysis also revealed the involvement of these targets in variety of cellular (74%), metabolic (70%), biological regulation (43%), multicellular organismal (35%), response to stimulus (33%), developmental (19%), immune system (8%) and localization (1%) processes.

**Fig. 7:**
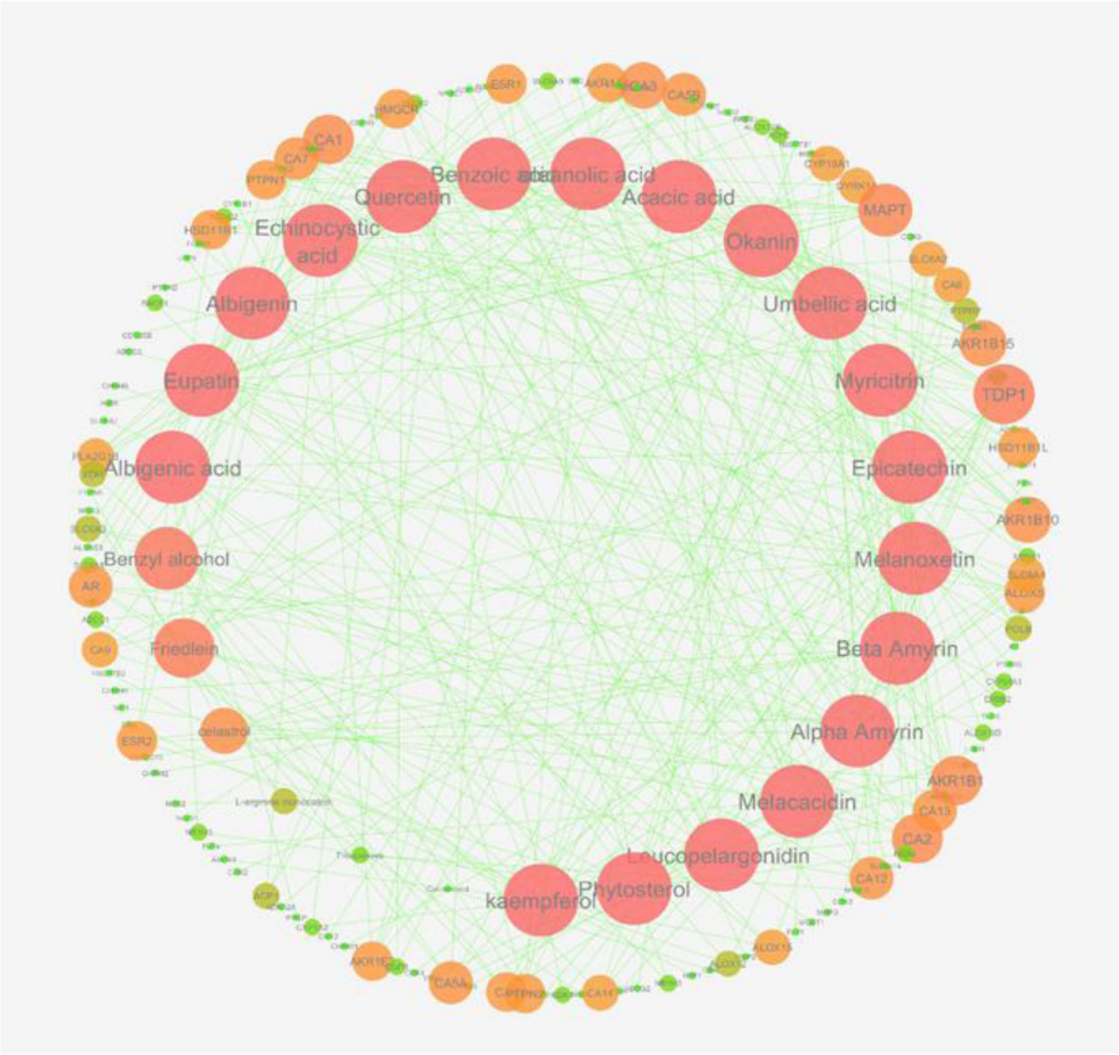
Global view of compound – target (C-T) network of 25 compounds interacting with 132 targets; The compounds are representing the inner clockwise degree-sorted circle, with nodes size and colour corresponding to degree of interactions with the targets nodes in outer circle. Higher degree of connectivity is implied as big red node as compare to small green node with lower degree of connectivity

The relationship between the compounds and targets was highlighted by the bipartite graph of compound – target (C-T) network of interaction (Fig. 7). The network was composed of 157 nodes (25 compounds and 132 targets) and 321 edges between them. The global view of C-T network represented the inner degree sorted circular layout of compounds connected to the outer circle of targets. The degree of interaction (i.e. edges) was consistent with the intensity of the color and size of the node. Higher degree of the interaction was represented by the superior node size and red color while lower degree with inferior node size and color reducing from orange to neon green. The degree sorted circular layout of compounds was consistent with anticlockwise sorting of compounds nodes with higher degree to lowest. The heterogeneity and centrality of network is 1.131 and 0.071 respectively, representing the biasness and centrality of nodes within the network. Consistently, the degree of some nodes indicated higher interactions as compared to other nodes. Most of the compounds nodes were active with higher degree of interactions. The nodes with higher degree of interaction are also called as hub that helps to understand the importance of compounds in therapeutic function of *Albizia lebbeck* (L.). Interestingly, the higher hit rate > 50% indicated the confidence of network to highlight the key components of *Albizia lebbeck* (L.). Consistently, among all the 25 compounds, 20 compounds have the degree higher than 10 with kaemferol (0.1243), phytosterol (0.1083) and okanin (0.1234) possessing highest betweenness centrality. However, topological co-efficient of melacacidin, L-arginine monocation, triterpenoids and quebrachitol was found to be zero that suggested the unique mechanism of action of these compounds as compared to synergistic behavior of other compounds. On the other hand, the tyrosyl – DNA phosphodiesterase 1 (TDP1) and microtubule-associated protein tau (MAPT) were found to be central targets in the interactome of *Albizia lebbck* (L.) compounds with 11 and 9 degree of interaction, respectively.

However, the protein-protein interaction or functional association of targets in STRING analysis enriched the 48 KEGG pathways. The detailed description of these pathways in tabulated along with their interacting proteins (Table 3). The major metabolic, PI3K-Akt signaling, Ras signaling and Rap1 signaling pathways were featured by the protein-protein interaction of 25, 17, 14 and 13 proteins, respectively.

**Table 3:** KEGG pathway enrichment and interacting proteins or targets

KEGG ID	pathway description	False discovery rate	Interacting proteins or targets
00910	Nitrogen metabolism	5.93e-21	CA1,CA12,CA13,CA14,CA2,CA3,CA4,CA5A,CA5B,CA6,CA7,CA9
04520	Adherens junction	3.81e-12	ACP1,EGFR,ERBB2,FGFR1,FYN,INSR,MET,PTPN1,PTPN6,PTPRF,SRC,YES1
04151	PI3K-Akt signalling pathway	6.47e-09	CDK4,CDK6,CHRM1,CHRM2,CSF1R,EGFR,FGFR1,FGFR2,FGFR3,FGFR4,FLT1,FLT4,INSR,KDR,KIT,MET,NOS3
04014	Ras signalling pathway	1.22e-08	CSF1R,EGFR,FGFR1,FGFR2,FGFR3,FGFR4,FLT1,FLT4,INSR,KDR,KIT,MET,PLA2G1B,PTPN11
04015	Rap1 signalling pathway	4.72e-08	CSF1R,EGFR,FGFR1,FGFR2,FGFR3,FGFR4,FLT1,FLT4,INSR,KDR,KIT,MET,SRC
04913	Ovarian steroidogenesis	5.27e-08	ALOX5,CYP17A1,CYP19A1,CYP1B1,HSD17B1,HSD17B2,INSR,LDLR
04144	Endocytosis	1.93e-07	CSF1R,EGFR,ERBB4,FGFR2,FGFR3,FGFR4,FLT1,KDR,KIT,LDLR,MET,SRC
00590	Arachidonic acid metabolism	2.04e-07	ALOX12,ALOX12B,ALOX15,ALOX15B,ALOX5,CYP2C19,PLA2G1B,PTGES
05206	MicroRNAs in cancer	1.05e-06	ABCB1,ABCC1,CDC25A,CDC25B,CDK6,CYP1B1,EGFR,ERBB2,FGFR3,MET
00140	Steroid hormone biosynthesis	1.44e-06	CYP17A1,CYP19A1,CYP1A2,CYP1B1,HSD11B1,HSD17B1,HSD17B2
01100	Metabolic pathways	2.81e-06	AKR1A1,AKR1B1,AKR1B10,ALOX12,ALOX12B,ALOX15,ALOX15B,ALOX5,AOX1,CYP17A1,CYP19A1,CYP1A2,CYP2C19,CYP51A1,GBA,HMGCR,HSD11B1,HSD17B1,HSD17B2,NOS1,NOS2,NOS3,PLA2G1B,PTGES,XDH
05200	Pathways in cancer	4.18e-06	AR,CDK4,CDK6,CSF1R,EGFR,ERBB2,FGFR1,FGFR2,FGFR3,KIT,MET,MMP2,NOS2
05205	Proteoglycans in cancer	4.18e-06	EGFR,ERBB2,ERBB4,ESR1,FGFR1,KDR,MET,MMP2,PTPN11,PTPN6,SRC
04810	Regulation of actin cytoskeleton	2.22e-05	CHRM1,CHRM2,CHRM4,CHRM5,EGFR,FGFR1,FGFR2,FGFR3,FGFR4,SRC
04020	Calcium signalling pathway	4e-05	CHRM1,CHRM2,CHRM5,EGFR,ERBB2,ERBB4,NOS1,NOS2,NOS3
05219	Bladder cancer	7.13e-05	CDK4,EGFR,ERBB2,FGFR3,MMP2
04976	Bile secretion	8.95e-05	ABCB1,ABCC2,ABCG2,CA2,HMGCR,LDLR
04726	Serotonergic synapse	0.000115	ALOX12,ALOX12B,ALOX15,ALOX15B,ALOX5,CYP2C19,SLC6A4
00591	Linoleic acid metabolism	0.000464	ALOX15,CYP1A2,CYP2C19,PLA2G1B
04915	Estrogen signalling pathway	0.000464	EGFR,ESR1,ESR2,MMP2,NOS3,SRC
04510	Focal adhesion	0.000734	EGFR,ERBB2,FLT1,FLT4,FYN,KDR,MET,SRC
04066	HIF-1 signalling pathway	0.000751	EGFR,ERBB2,FLT1,INSR,NOS2,NOS3
04080	Neuroactive ligand-receptor interaction	0.000844	ADRA2A,ADRA2B,ADRA2C,CHRM1,CHRM2,CHRM4,CHRM5, NR3C1,PRSS1
04725	Cholinergic synapse	0.000851	ACHE,CHRM1,CHRM2,CHRM4,CHRM5,FYN
05218	Melanoma	0.000996	CDK4,CDK6,EGFR,FGFR1,MET
05204	Chemical carcinogenesis	0.0011	CYP1A2,CYP1B1,CYP2C19,HSD11B1,MGST1
02010	ABC transporters	0.00138	ABCB1,ABCC1,ABCC2,ABCG2
05215	Prostate cancer	0.00239	AR,EGFR,ERBB2,FGFR1,FGFR2
00232	Caffeine metabolism	0.00402	CYP1A2,XDH
05223	Non-small cell lung cancer	0.00402	CDK4,CDK6,EGFR,ERBB2
05212	Pancreatic cancer	0.00619	CDK4,CDK6,EGFR,ERBB2
00982	Drug metabolism - cytochrome P450	0.00678	AOX1,CYP1A2,CYP2C19,MGST1
05120	Epithelial cell signalling in Helicobacter pylori infection	0.0074	EGFR,MET,PTPN11,SRC
00980	Metabolism of xenobiotics by cytochrome P450	0.00805	CYP1A2,CYP1B1,HSD11B1,MGST1
04917	Prolactin signaling pathway	0.00873	CYP17A1,ESR1,ESR2,SRC
00040	Pentose and glucuronate interconversions	0.01	AKR1A1,AKR1B1,AKR1B10
04010	MAPK signalling pathway	0.01	CDC25B,EGFR,FGFR1,FGFR2,FGFR3,FGFR4,MAPT
04060	Cytokine-cytokine receptor interaction	0.0119	CSF1R,EGFR,FLT1,FLT4,KDR,KIT,MET
00380	Tryptophan metabolism	0.0151	AOX1,CYP1A2,CYP1B1
04012	ErbB signalling pathway	0.0151	EGFR,ERBB2,ERBB4,SRC
04022	cGMP-PKG signalling pathway	0.0256	ADRA2A,ADRA2B,ADRA2C,INSR,NOS3
05202	Transcriptional mis-regulation in cancer	0.03	CSF1R,FLT1,MET,MMP3,MPO
00561	Glycolipid metabolism	0.0349	AKR1A1,AKR1B1,AKR1B10
00330	Arginine and proline metabolism	0.0397	NOS1,NOS2,NOS3
04370	VEGF signalling pathway	0.0418	KDR,NOS3,SRC
04964	Proximal tubule bicarbonate reclamation	0.0418	CA2,CA4
05214	Glioma	0.0449	CDK4,CDK6,EGFR
04110	Cell cycle	0.0483	CDC25A,CDC25B,CDK4,CDK6

**Fig. 8:**
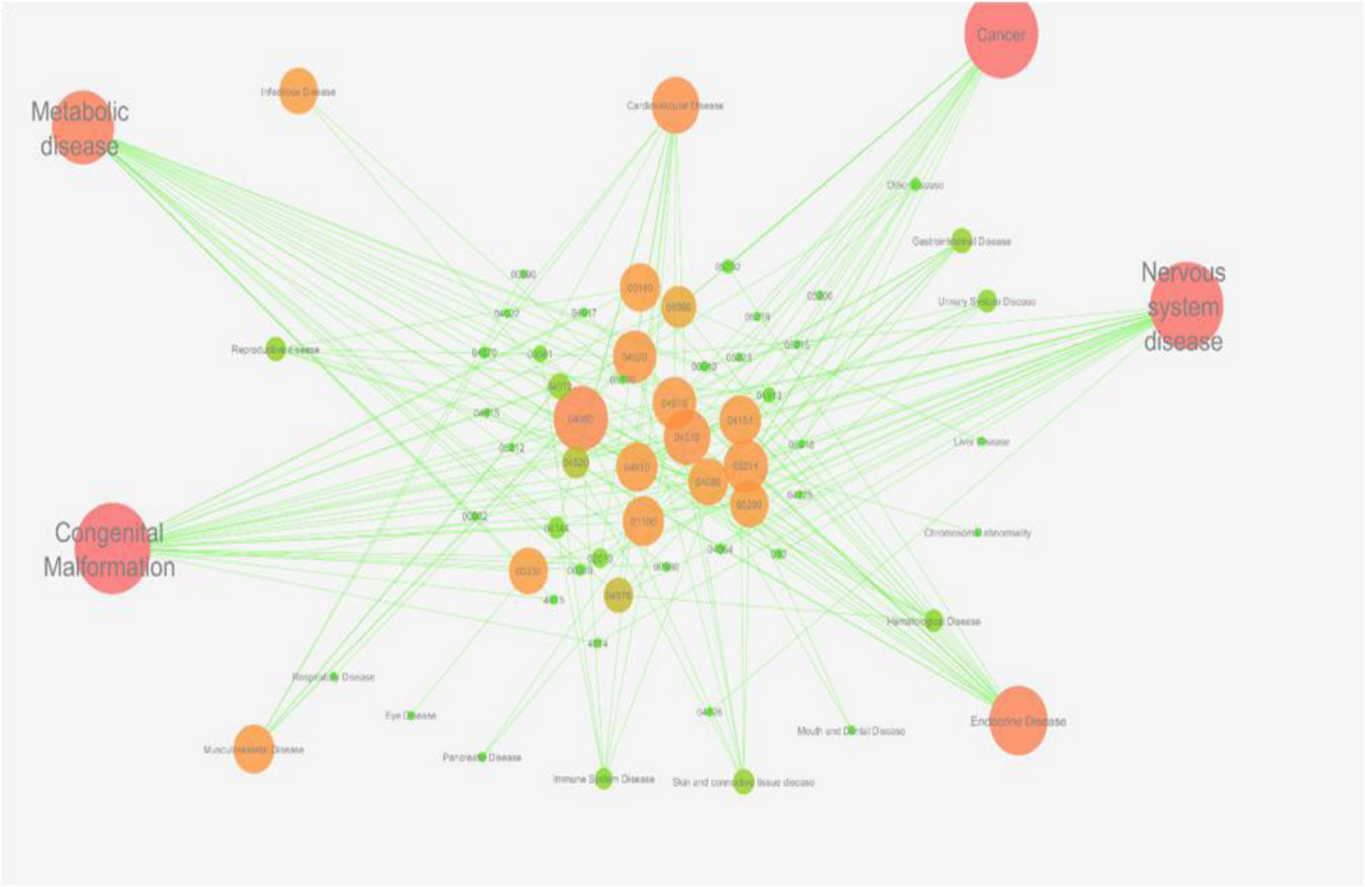
Global view of Pathway – Disease system (P-D) network; Bipartite graph representing the pathways as central nodes coloured and sized by their connectivity to peripheral nodes of disease system at organ level. Higher degree of connectivity is implied as big red node as compare to small green node with lower degree of connectivity

A bipartite graph was constructed to highlight the relationship of these pathways to the development of diseases at organ level (Fig. 8). This network was made of 64 nodes interacting each other with 167 edges. Peripheral nodes highlighted the disease system while central nodes represented the pathways. The size and color of nodes represented the degree of interaction as defined previously. The network centralization and heterogeneity were found to be 0.293 and 0.972 respectively, indicating the biasness and centrality among nodes. The nodes of congenital malformation, nervous system disease and metabolic disease were found to have high node degree and interacted with of 24, 21 and 21 pathways, respectively. This degree of interaction was further followed by the nodes of endocrine disease, cancer, cardiovascular and musculoskeleton disease with their interaction with 17, 14, 12 and 6 pathways, respectively.

## Discussion

This study was aimed to employ the experimental and system’s pharmacology approach to validate the traditional use and investigate the mechanism of action of *Albizia lebbeck* (L.) in Parkinson’s disease (PD). In this study, haloperidol (1 mg/kg) was intraperitoneally administered for 21 days to induce the cataleptic feature of PD. Haloperidol is a neuroleptic drug that acts primarily through dopamine receptor subtype D2 and produce the catalepsy as a extrapyramidal side effect [37]. The haloperidol induced catalepsy serve as a robust model to investigate the anti-Parkinsonian activity [38]. The catalepsy is referred to a state where animals are unable to correct their imposed postures due to motor dysfunctions [39]. The HPD group was found to be highly cataleptic as demonstrated with higher duration of catalepsy at all intervals that was consistent with literature [23]. However, the co-treatment with ALE 100, 200 and 300 significantly reduced the catalepsy in a dose dependent manner that was comparable STD. Moreover, the motor coordination and muscular strength was further assessed with the narrow beam and hang test. The HPD group revealed the lowest time latency in hang test and highest time latency to walk through the beam. However, the ALE – coadministration improved the globular muscle strength and coordination thereby reversing the motor dysfunction of Parkinson’s. In addition to this, locomotor activity and exploratory activity of experimental rats was also assessed in open field test to address the cognitive and motor dysfunction associated with the Parkinson’s. This test works on the principle that rats with lower cognition, skeleton muscles strength and endurance shows lower locomotor activity and exploratory behavior [27]. The HPD group revealed the marked reduction in horizontal exploration and locomotor activity as compare to STD groups. However, the ALE co-administration improved the locomotor and exploratory activity suggesting its role in improving the motor dysfunction of Parkinson’s. Together, these behavior studies suggested the overall improvements in motor function of experimental groups treated with ALE.

In addition to its cataleptic function, the haloperidol has also been implicated as potential source of oxidative stress [40]. Interestingly, dopamine can also be metabolized to free radicals by the monoamine oxidase (MAO) or auto-oxidation in PD [41]. These reactive metabolites of dopamine can form the cysteinyl adducts at substantia nigra of PD patient thus induces the neurodegeneration [42]. Moreover, the susceptibility of α-synuclein to reactive metabolites can also activates the microglia that further fuel the neurotoxicity [43]. In our study, the endogenous antioxidant enzymes and GSH content in the brain tissue of HPD group was severely compromised that showed acute oxidative stress consistent with previous studies [23]. Superoxidse dismutase (SOD) is a key endogenous antioxidant enzyme that has been found to be depressed in the PD and results into the neuronal load of O_2_• free radicals [44]. Moreover, the activity of catalase (CAT) was also found to be compromised in PD that suggests the production of OH• free radicals by Fenton type reaction in neurons thus contributing to the neurodegeneration in PD. Additionally, the content of GSH is depleted along the saturation of antioxidant enzyme [45]. However, the ALE co-administration significantly recovered the GSH content and activity of these enzymes thereby supported the therapeutic potential of ALE to mitigate the oxidative stress in PD. These results were further corroborated with histological studies that revealed the ALE potential to ameliorate the lipid peroxidation and neurodegeneration while reversing the tissue architecture of PD. Therefore, together these biochemical studies supported the functional outcomes of behavioral studies and advocated the therapeutic potential of ALE in the treatment of PD.

Plants are the rich source of diverse bioactive chemical species that determines the medicinal properties of them. The multi-component system and synergism between them makes the plants challenging source to probe the potential bioactive compounds responsible for their pharmacological activities. Interestingly, the virtue of synergism is complementing the emerging approach of “multi-drugs and multi-targets” with the fall of “one-drug and one target” approach due to dynamic biological systems [46]. In this context, an integrate approach of system’s pharmacology has been emerged that considers the synergism of phytochemicals to approximate their pharmacological properties with potential targets in a dynamic biological system [47]. The holistic approach of systems pharmacology to integrate the genomics, proteomics, metabolomics and bioinformatics provides the exceptional platform to study the essence of synergism among the phytochemicals to treat complex diseases. Therefore, we adopted this approach to delineate the *Albizia lebbeck* (L.) mechanism of action in PD. In the present study, the 86% of total 30 compounds were filtered by the Lipinski rule of five for druglikeness in SwissADME. Lipinski rule of five describe the relationship between physicochemical properties and pharmacokinetics by defining the physicochemical benchmarks to predict the orally active compounds [32]. These compounds were further mapped to predict the 132 targets by reverse pharmacophore modelling in Swiss Target Prediction tool. The reverse pharmacophore approach computes the 2D and 3D pharmacophore of targets with known ligands and predict them by the measure of structural similarity [33]. The class of these targets or proteins was distinguished and relevance to biological functions was determined by PANTHER classification system. These targets were predominantly classified as oxidoreductase, receptors (G-Protein coupled), hydrolases, and nucleic acid binding proteins, transporter, transcription factors and transferases. The relevance in variety of cellular, metabolic, biological regulation, multicellular organismal, response to stimulus, developmental, immune system and localization processes suggested the vitality of these proteins in biological system. The compound-target (C-T) network explained that most of the compounds actively involved in the synergistic pattern of interaction with targets. This network also indicated that many of the compounds were interacting with more than one target and vice versa. Therefore, synergistic modulation of series of the targets by number of compounds suggested the “multi-drugs and multi-targets” as a essence to the mechanism of action of *Albizia lebbeck* (L.). It is generally accepted that compounds or targets with the higher degree of interaction are more pharmacologically crucial [48]. Degree is a basic topological parameter in network analysis that determine the importance of compounds and targets [49]. Betweenness centrality is another important topological parameter that determine the significance of node location within the network [50]. In our network, we found the correlation between the degree and betweenness centrality as compounds, such as kaemferol, phyosterol and okanin, demonstrated both the higher degree of interaction and betweenness centrality thus represented as key players of *Albizia lebbeck* (L.). The tyrosyl – DNA phosphodiesterase 1 (TDP1) and microtubule-associated protein tau (MAPT) were the central nodes amongst the targets with highest degree of interaction thus represented as the targets modulated by majority of *Albizia lebbeck* (L.). TDP1 is an DNA repair enzyme that has been found to catalyze breakage of the covalently linked oxidative stress induced DNA adducts at 3′- phosphate end and implicated as a target of various anticancer drugs [51]. It has been found to repair the oxidative stress induced single strand breaks (SSB) in astrocytes and loss of TDP1 function has also been implicated in development of progressive age-related cerebellar atrophy that highlighted its involvement in neural homeostasis [52]. MAPT encodes the tau protein that has been implicated to regulate the axonal and micro tubular function. Moreover, single nucleotide polymorphism (SNP) at MAPT has been associated with tauopathies in development of neurodegenerative disease like Alzheimer’s and Parkinsonism [53]. There are also evidences that suggest the risk associated with MAPT haplotypes in the pathogenesis of PD [54, 55]. The higher centrality or degree of interaction of these target nodes in C-T network of *Albizia lebbeck* (L.) represent them the key player of its mechanism of action in PD. Furthermore, the functional association of the targets in STRING analysis enriched the 48 KEGG pathways. The neuroactive ligand receptor pathway was found to be in top ten overrepresented pathway in the genome-wide association studies (GWAS) of PD [56]. The experimental evidence had suggested the association of neuroactive ligand receptor pathway in α-synuclein toxicity in the pathogenesis of PD [57]. The serotoninergic synapses has been implicated as increase post-synaptic density of serotonin receptors (5HT_1A_, 5HT_2A_) in neocortex of PD patients [58]. A study has suggested the degeneration of serotonergic terminals is an early process in the pathogenesis of PD and with preferential lower rate of degeneration as compare of dopaminergic neurons [59]. The aberrant spread of serotonergic terminals in putamen, non-physiologically converts the exogenous levodopa and results into higher swings of dopamine that develops the levodopa associated dyskinesia [60]. The cholinergic and dopaminergic neurons are highly conserved in the striatum and extensively interact bidirectionally to regulate the motor coordination, cognitive function and rewards [61]. Cholinergic synapse has been found to induce glutamatergic and GABAergic plasticity and increase the Ach at striatum that induce the levodopa associated dyskinesia in PD [62]. Various lines of evidences have also suggested the MAPK signaling in regulation of autophagy and neurodegeneration in PD [63]. Therefore, these pathways further highlighted the functional association of interactome of *Albizia lebbeck* (L.) targets to the PD. However, all of the KEGG pathways were further indexed to KEGG disease database and Pathway – Disease (P-D) network was constructed to explore the therapeutic space of *Albizia lebbeck* (L.). The global view of network revealed that these pathways mainly interacted with the metabolic, endocrine, cancer, cardiovascular, nervous system diseased nodes which was consistent with the majority of *Albizia lebbeck* (L.) traditional uses [8]. Among all the disease nodes, we found that nervous system diseases node shared the highest degree of interaction with 21 pathways after the node of congenital malformation which interacted with 24 pathways. Therefore, these insights of *Albizia lebbeck* (L.) systems pharmacology were found to be consistent with in-vivo experimental results of anti-Parkinsonian activity and provided the deep understanding regarding its mechanism of action in PD.

## Conclusion

The in-vivo experiment for anti-Parkinson’s activity of *Albizia lebbeck* (L.) concluded that *Albizia lebbeck* (L.) improved the motor functions and reversed the biochemical damages in brain tissue of PD. The systems pharmacology approach in this study investigated the mechanism of action of the *Albizia lebbeck* (L.) in PD and clearly suggested the synergistic effect of its phytochemicals in the development of its therapeutic effect. This approach supported the theory of “multi-drug and multi target” and indicated the therapeutic efficiency of *Albizia lebbeck* (L.) for nervous system diseases. However, the networks also highlighted the therapeutic efficiency for other disease system that should also be deciphered experimentally. Therefore, the present work served as the alternative strategy to provide the deep understanding of therapeutic behavior and sophistically validates the traditional uses of *Albizia lebbeck* (L.) to improve the efficiency of drug discovery.

## Data Availability

The datasets used and/or analysed during the current study available from the corresponding author on reasonable request.

## Abbreviations

5HT1: Serotonin 1A receptor
5HT2A: Serotonin 2A receptor
ABCB1: Multidrug resistance protein 1
ABCC1: Multidrug resistance-associated protein 1
ABCC2: Canalicular multispecific organic anion transporter 1
ABCG2: ATP-binding cassette sub-family G member 2
ACHE: Acetylcholinesterase
ACP1: Low molecular weight phosphotyrosine protein phosphatase
ADRA2A: Alpha-2A adrenergic receptor
ADRA2B: Alpha-2B adrenergic receptor
ADRA2C: Alpha-2C adrenergic receptor
AKR1A1: Alcohol dehydrogenase [NADP(+)]
AKR1B1: Aldo-keto reductase family 1 member B1
AKR1B10: Aldo-keto reductase family 1 member B10
Akt: Protein kinase B
ALE 100: 100 mg/kg treatment control
ALE 200: 200 mg/kg treatment control
ALE 300: 300 mg/kg treatment control
ALE: Albizia lebbeck (L.) extract
ALOX12: Arachidonate 12-lipoxygenase, 12S-type
ALOX12B: Arachidonate 12-lipoxygenase, 12R-type
ALOX15: Arachidonate 15-lipoxygenase
ALOX15B: Arachidonate 15-lipoxygenase B
ALOX5: Arachidonate 5-lipoxygenase
AOX1: Aldehyde oxidase
AR: Androgen receptor
BT: Tissue homogenate of brain
CA1: Carbonic anhydrase 1
CA12: Carbonic anhydrase 12
CA13: Carbonic anhydrase 13
CA14: Carbonic anhydrase 14
CA2: Carbonic anhydrase 2
CA3: Carbonic anhydrase 3
CA4: Carbonic anhydrase 4
CA5A: Carbonic anhydrase 5A
CA5B: Carbonic anhydrase 5B
CA6: Carbonic anhydrase 6
CA7: Carbonic anhydrase 7
CA9: Carbonic anhydrase 9
CAT: Catalase
CDC25A: M-phase inducer phosphatase 1
CDC25B: M-phase inducer phosphatase 2
CDK4: Cyclin-dependent kinase 4
CDK6: Cyclin-dependent kinase 6
cGMP: Cyclic guanosine monophosphate
CHRM1: Muscarinic acetylcholine receptor M1
CHRM2: Muscarinic acetylcholine receptor M2
CHRM4: Muscarinic acetylcholine receptor M4
CHRM5: Muscarinic acetylcholine receptor M5
CSF1R: Macrophage colony-stimulating factor 1 receptor
C-T network: Compound - Target network
CYP17A1: Steroid 17-alpha-hydroxylase/17,20 lyase
CYP19A1: Cytochrome P450 19A1
CYP1A2: Cytochrome P450 1A2
CYP1B1: Cytochrome P450 1B1
CYP2C19: Cytochrome P450 2C19
CYP51A1: Lanosterol 14-alpha demethylase
DF: Dilution factor
DNA: Deoxyribonucleic acid
DTNB: 5,5′-Dithiobis-(2-nitrobenzoic acid)
EGFR: Epidermal growth factor receptor
ERBB2: Receptor tyrosine-protein kinase erbB-2
ERBB4: ERBB4 intracellular domain
ESR1: Estrogen receptor
ESR2: Estrogen receptor beta
FGFR1: Fibroblast growth factor receptor 1
FGFR2: Fibroblast growth factor receptor 2
FGFR3: Fibroblast growth factor receptor 3
FGFR4: Fibroblast growth factor receptor 4
FLT1: Vascular endothelial growth factor receptor 1
FLT4: Vascular endothelial growth factor receptor 3
FYN: Tyrosine-protein kinase Fyn
GBA: Glucosylceramidase
GSH: Glutathione
GWAS: Genome-wide association studies
H&E: Hematoxylin and Eosin
H2O2: Hydrogen Peroxide
HIF-1: Hypoxia-inducible factor 1
HMGCR: 3-hydroxy-3-methylglutaryl-coenzyme A reductase
HPD: Disease control
HSD11B1: Corticosteroid 11-beta-dehydrogenase isozyme 1
HSD17B1: Estradiol 17-beta-dehydrogenase 1
HSD17B2: Estradiol 17-beta-dehydrogenase 2
IMPPAT: Indian Medicinal Plants, Phytochemistry and Therapeutics
INSR: Insulin receptor
KDR: Vascular endothelial growth factor receptor 2
KIT: Mast/stem cell growth factor receptor Kit
LDLR: Low-density lipoprotein receptor
MAO: Monoamine oxidase
MAPK: Mitogen-activated protein kinase
MAPT: Microtubule-associated protein tau
MET: Hepatocyte growth factor receptor
MGST1: Microsomal glutathione S-transferase 1
MMP2: Matrix metalloproteinase-2
MMP3: Stromelysin-1
MPO: Myeloperoxidase
NC: Normal control
NOS1: Nitric oxide synthase 1
NOS2: Nitric oxide synthase 2
NOS3: Nitric oxide synthase 3
NR3C1: Glucocorticoid receptor
P-D: Network Pathway - Disease network
PD: Parkinson’s disease
PI3K: Phosphoinositide 3-Kinase
PKG: Protein kinase G
PLA2G1B: Phospholipase A2
PRSS1: Alpha-trypsin chain 1
PTGES: Prostaglandin E synthase
PTPN1: Tyrosine-protein phosphatase non-receptor type 1
PTPN6: Tyrosine-protein phosphatase non-receptor type 6
PTPRF: Receptor-type tyrosine-protein phosphatase F
Rap1: Ras-related protein 1
SEM: Standard error of the mean
SLC6A4: Sodium-dependent serotonin transporter
SNP: Single neucleotide polymorphism
SOD: Superoxide dismutase
SRC: Proto-oncogene tyrosine-protein kinase Src
SSB: Single strand break
STD: Standard control
TCA: Trichloroacetic acid
TDP1: Tyrosyl DNA phosphodiesterase 1
VEGF: Vascular endothelial growth factor
VU: Volume of aliquot
XDH: Xanthine dehydrogenase/oxidase
YES1: Tyrosine-protein kinase Yes
δOD: Changing absorbance / minute
